# Fair Workweek laws in the US: An appraisal of intended and unintended consequences

**DOI:** 10.1126/sciadv.aea8632

**Published:** 2026-06-24

**Authors:** Daniel Schneider, Kristen Harknett, David Arbelaez

**Affiliations:** ^1^ Harvard Kennedy School, Cambridge, MA, USA.; ^2^ University of California, San Francisco, San Francisco, CA, USA.

## Abstract

Fair Workweek laws, implemented in several large cities and one state, represent a new wave of innovation in a century of contestation and legislation around work time. Where previous legislation sought to regulate overwork, these laws aim to regulate work schedule instability and insufficiency of work hours, but evidence on their effects is incomplete. Using stacked difference-in-differences models and original survey data, we find robust evidence that Fair Workweek regulations led to increases in advance notice of schedules and time to rest between shifts and reductions in last-minute schedule changes. We find no evidence of compensatory retrenchment in other aspects of job quality. These average effects disguise substantial heterogeneity across jurisdictions, pointing to the importance of local enforcement in realizing policy goals. Fair Workweek legislation has realized some intended effects on work schedules without unintended consequences, but the potential for more widespread, effective enforcement has not been fully realized.

## Teaser

Fair Workweek laws help to reduce work schedule instability and unpredictability without negative unintended consequences.

## INTRODUCTION

More than 150 years ago, labor advocates demanded a radical reform to employment relations—an eight-hour workday. Passed nearly 60 years later, the Fair Labor Standards Act (FLSA) saw the realization of some of labor’s early demands, including a 40-hour week. The centrality of work time shaped early labor movements around the world, and work time remains a central concern of both labor and social scientists focused on work, inequality, and wellbeing (*1*–*3*).

However, the nature of work has changed in America in fundamental ways since the FLSA was enacted, especially since the 1980s, with employment becoming more precarious on multiple dimensions (*4*) and with large institutional actors, such as employers, effectively shifting risk from organizations to households (*5*). Ongoing conflicts over working time are on vivid display in the contemporary American service sector, where hourly workers in restaurants and retail face not only low wages and limited benefits but also the twinned challenges of work schedules that are unstable and unpredictable and work hours that are often insufficient (*6*, *7*). Rather than work a 9-to-5 or even a regular night shift, workers contend with schedules that vary from day-to-day and week-to-week, often with little advance notice and subject to frequent last-minute changes (*7*, *8*). Where workers once sought freedom from overwork, today’s cashiers and cooks scrounge for sufficient hours to get by (*9*). These twin challenges appear to produce a destructive chain reaction—sparking family chaos (*10*–*12*) and burning through economic and health reserves (*13*, *14*).

Federal labor standards have not kept up with this new precarious reality. However, over the past decade, localities and states have stepped in to regulate this new world of unstable, unpredictable, and insufficient work scheduling. “Fair Workweek” (FWW) or “Predictable Scheduling” ordinances have been passed in 10 jurisdictions, including Seattle, New York City (NYC), Chicago, Philadelphia, and Oregon.

These labor standards follow a common template. They seek to address work scheduling by requiring large-chain service sector employers to provide at least 2 weeks’ notice of work schedules, imposing predictability pay requirements for last-minute changes or on-call work, and regulating back-to-back closing then opening shifts, also known as “clopening” shifts. In addition, these standards aim to increase workers’ access to a sufficient number of work hours by requiring employers to offer newly available shifts to incumbent workers before hiring new part-time workers. Similar legislation has been introduced in the US House and Senate, considered in the UK, and adopted by the European Council.

FWW standards have been met with vociferous opposition, often from business groups (*15*–*17*). While prior research provides evidence that more stable and predictable scheduling could improve business outcomes by increasing productivity and reducing turnover (*18*, *19*), it is also possible that employers would perceive compliance with these mandates to be costly (*20*).

In response, employers might then compensate for compliance with FWW provisions through other cost-reducing channels of adjustment, such as by reducing wages or by reducing fringe benefits (*21*). It is also possible that the protections afforded by FWW laws could lead to changes in employer hiring and retention practices and/or worker application and turnover decisions. If the protections, for instance, served to attract more skilled or experienced workers to the covered jobs, then any costs of FWW compliance could be offset by increases in worker productivity rather than through channels of adjustment.

These FWW laws are innovative, but the ability of firms to understand the complex regulations and the capacity of local regulators to effectively enforce scheduling standards may be limited (*20*). In the face of substantial resource constraints (*22*), rather than proactively (or even randomly) investigate worksites, most enforcement follows a complaint-based model (*23*), in which workers must be aware of their rights and feel sufficiently empowered to come forward to bring a complaint when they suspect violation. In the case of FWW laws, local enforcement agencies must then have the capacity to conduct sophisticated analysis of company time-clock and scheduling records to assess compliance and violations (*24*). For these reasons, the efficacy of FWW laws in achieving their intended effects is uncertain a priori.

The importance of local regulators to the successful implementation of FWW laws also opens up the possibility of variation in the efficacy of these laws across jurisdictions. Cities and states vary widely in their resources and capacities for informing workers and employers about the new rights and requirements stipulated by these laws, investigating worker complaints, and imposing penalties on employers for violations (*25*, *26*). These differences may lead to treatment effect heterogeneity by place. Heterogeneous treatment effects by group or place are commonplace in causal research when tested for (*27*, *28*), and revealing this heterogeneity is important for advancing understanding and generating theory about the conditions under which interventions are most effective (*27*). Systematic data on enforcement capabilities by jurisdiction is not readily available, but one measure of the strength of enforcement is public settlements for FWW violations. By that metric, New York City and their Department of Consumer and Worker Protection (DCWP) stands out as having the largest and most high-profile settlements for violations of the city’s FWW Law (*29*, *30*). These large settlements were the culmination of multiyear investigations involving multiple locations and thousands of workers, a strong indicator of DCWP’s organizational capacity around FWW enforcement (*30*, *31*).

Currently, our knowledge about the effectiveness of FWW laws with respect to schedule stability and hours sufficiency, about channels of adjustment, and about heterogeneity in effectiveness across jurisdictions is incomplete. On the one hand, five studies conduct law-specific case-study evaluations. These studies each focus on a single FWW ordinance, marshaling creative sources of data to rise to the challenge of estimating the effects of specific ordinances. Harknett *et al.* (*32*) focus on Seattle’s ordinance and draw on survey data from workers at dozens of large firms from 1 year before and up to 2 years after implementation to document increases in the share of workers receiving at least 2 weeks’ notice and reductions in last-minute shift changes, but find no effects on amount of work hours. Ananat *et al.* (*33*) estimate the effects of Emeryville, CA’s ordinance using a daily text survey of 78 working parents and find reductions in schedule instability, with no effects on number of work hours. Petrucci *et al.* (*34*) draw on 98 in-depth interviews with workers in Oregon to conclude that the law was broadly ineffective. Two other studies estimate the effects of the Oregon and NYC laws but only on downstream labor market outcomes, not on scheduling. Focusing on Oregon, Gruber (*35*) draws on data from the Quarterly Workforce Indicators and American Community Survey, which allow her to estimate overall null effects of the law on employment and earnings, with some evidence of heterogenous effects by gender. Pickens and Sojourner (*36*) draw on data from the Quarterly Census of Employment and Wages (QCEW) to estimate the effects of New York City’s FWW law on employment and find no effects, although, similar to Gruber (*35*), they cannot discern whether this is because of minimal employer adjustment to meaningful changes in scheduling or simply minimal compliance with the law. In sum, these studies suggest that FWW laws in Seattle and Emeryville may have improved work scheduling and that laws in those two jurisdictions and Oregon and New York City did not have major unintended effects on employment and/or number of work hours.

On the other hand, three studies look across multiple jurisdictions. Kwon and Raman (*37*) aggregate across three FWW jurisdictions using harmonized administrative data from a scheduling software vendor. This work also finds increases in advance notice and no evidence of reductions in hiring, work hours, or increases in the use of part-time workers. However, the Kwon and Raman (*37*) sample is composed of data from just three employers, limiting external validity. Yelowitz (*38*) draws on the Current Population Survey (CPS) to measure the effects of four FWW laws, reporting increases in involuntary part-time work. However, Yelowitz (*38*) is limited in his ability to correctly identify treated and control workers. Last, Lambert *et al.* (*39*) field an original survey of 1800 hourly workers in covered jobs within and just outside of the city limits of three jurisdictions with FWW ordinances—Chicago, Seattle, and New York City. Estimating single differences without a pretreatment comparison, they find evidence of significantly more advance notice among covered workers and of more extra pay for clopening shifts and last-minute timing changes, with no apparent impacts on access to hours or canceled shifts. Unlike prior studies (*32*, *33*, *37*), Lambert *et al.* (*39*) do not examine changes in the incidence of practices beyond notice, only compensation. Furthermore, the lack of pretreatment observation makes it difficult to interpret the reported effects.

In all, these widely varying methodologies, data sources, and cases represent pragmatic and creative adaptions to serious challenges of data availability and consistency. Some clear themes emerge from these findings. The four quantitative studies (*32*, *33*, *37*, *39*) that have looked at scheduling outcomes all find an increase in advance notice. The evidence is more mixed on downstream labor market outcomes, with some work finding null effects on hours (*32*, *33*, *37*), other work finding both large increases in involuntary part-time employment (*38*) and no effects (*37*), two studies finding null employment effects (*36*, *37*), and lastly, one study finding heterogenous effects on hours and employment (*35*). However, it is difficult to generalize about the effectiveness and effects of FWW standards on scheduling and on potential channels of adjustment given the necessarily varied data sources and methodological approaches of these studies.

We draw on original data that we collected over the course of 7 years and 14 repeated cross-sectional survey waves from more than 87,000 hourly workers in the service sector across the United States. We designed our survey to estimate the effects of FWW laws after realizing that early ordinances were likely to precede a wave of policy making. Our sampling was designed then to target exactly the covered population of workers (workers at large-chain firms in the retail and food service sectors) and included oversamples to ensure sufficient sample sizes as Seattle, Oregon, New York City, Philadelphia, and Chicago passed FWW laws. We developed and included novel measures of work scheduling collaboratively with labor regulators, designed to capture compliance along with other measures of job quality to capture potential channels of adjustment.

We use these data to estimate stacked difference-in-differences (DiD) models that align pretreatment and posttreatment observations with the timing of each of five ordinances’ implementation (Seattle, Oregon, New York City, Chicago, and Philadelphia), including individual-level and area-level controls. We describe the provisions of each of these five ordinances, as well as those of five other ordinances that have been passed, but for which we lack sufficient data to estimate effects, in table S6. We estimate the first-order effects of the ordinances on scheduling outcomes and hours, then effects on potential channels of adjustment including wages and fringe benefits, and lastly, examine heterogeneity in the efficacy of FWW standards across these five jurisdictions.

## RESULTS

### Effects of FWW laws on scheduling and hours

Table 1 presents results from stacked DiD models that estimate the effects of FWW laws on a set of work schedule outcomes. Model 1 presents stacked DiD estimates without additional covariates but with the full set of year- and employer-by-subexperiment fixed effects (FEs). Model 2 adds a set of individual-level controls, and model 3 adds area-level controls.

**Table 1.** Effects of FWW laws on scheduling and work hours. +*P* < 0.10. *P < 0.05. **P < 0.01. ***P < 0.001. Weighted using equal subexperiment weights. SEs clustered at geographic level. Pretreatment treatment group mean in parentheses. Avg., average.

	(1)	(2)	(3)
Two weeks’ notice (μ = 0.45)			
FWW × post	0.133***	0.133***	0.130***
	(0.030)	(0.031)	(0.032)
Clopening (μ = 0.45)			
FWW × post	−0.079*	−0.084***	−0.084***
	(0.031)	(0.025)	(0.025)
Timing change (μ = 0.65)			
FWW × post	−0.061*	−0.064*	−0.067*
	(0.028)	(0.027)	(0.029)
Canceled shift (μ = 0.15)			
FWW × post	−0.023	−0.024	−0.026
	(0.022)	(0.021)	(0.021)
On-call shift (μ = 0.24)			
FWW × post	−0.008	−0.011	−0.013
	(0.022)	(0.021)	(0.019)
Usual hours (μ = 33.6)			
FWW × post	−0.552	−0.501	−0.373
	(0.675)	(0.727)	(0.723)
*N*	285,993	285,993	285,993
Avg. subexperiment *N*	57,199	57,199	57,199
Year × subexperiment FE	X	X	X
Employer × subexperiment FE	X	X	X
Individual-level controls		X	X
Area-level controls			X

Among the measures of work schedules, FWW regulations had the largest effects on workers receiving at least 2 weeks of advanced notice of their work schedule, shown in the first row of Table 1. FWW regulations increased advance notice by 13 percentage points (p.p.) across all models. In percentage terms, against a pretreatment treatment group mean of 45% receiving 2 weeks’ notice, these new regulations increased schedule notice by about 29%.

Table 1 also shows that FWW regulations reduced the experience of working back-to-back closing then opening shifts (clopenings), with little time for rest in between. After FWW laws went into effect, workers covered by these laws experienced an 8-p.p. reduction in clopening shifts (models 2 and 3) compared with their counterparts not covered by these new laws, a roughly 19% change compared to a pretreatment treatment group mean of 45%. FWW laws also reduced last-minute timing changes to work schedules by about 6 p.p., a roughly 10% change compared to the 65% mean.

Although employers are required to provide extra compensation to workers for canceled shifts, we find that FWW laws had no effect on shift cancelations or on-call shifts. Those in covered and uncovered locations experienced a similar trend in both of those scheduling outcomes after FWW laws took effect. The null effects on shift cancelations and on-call shifts may be explained by implementation research, which has found confusion on the part of managers about when shift changes were permissible without extra pay (*40*) and that employers exploited this ambiguity to avoid compliance (*34*).

The access to hours provision of FWW laws was intended to increase work hours for involuntary part-time workers, but we find no evidence of hour increases for covered workers. We estimate that FWW laws reduced weekly work hours by a little less than 0.6 hours per week in model 1, but that effect was attenuated to 0.37 hours and statistically insignificant with the addition of area-level controls in model 3. Against average usual weekly work hours of 33, this represents a 1.1% reduction.

**Fig. 1.**
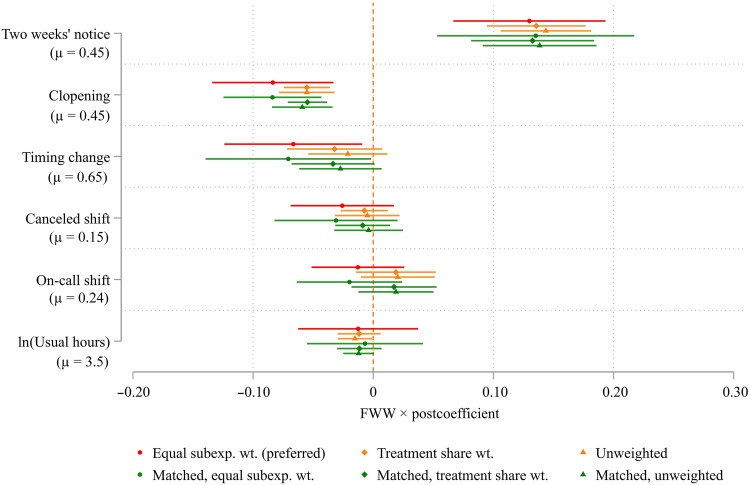
Robustness of main effects of FWW laws to alternative matching and weighting. SEs clustered at geographic level. Matching: coarsened exact matching (CEM) between treatment and control observations on basis of demographic and work characteristics. All models include year × subexperiment FEs, employer × subexperiment FEs, individual-level controls, and area-level controls. Error bars represent 95% confidence intervals. *N* = 285,993 for non-CEM models and *N* = 167,775 for CEM models. subexp. wt., subexperiment weights. Pretreatment treatment group mean in parentheses.

Figure 1 (and in table S1) shows the robustness of our model 3 estimates to variations in the weighting and comparison-group matching approaches. The positive effects of the FWW laws on receiving at least 2 weeks’ advance notice of work schedules and on clopening shifts are robust to all model permutations—both in terms of magnitude and statistical significance. The results for timing changes are somewhat more sensitive, with the estimates somewhat smaller in magnitude and nonsignificant when using alternative weights. The estimates of the laws’ null effects on on-call shifts and canceled shifts, as well as on hours, were not sensitive to matching approach or weighting.

We also assess robustness of our results to several other alternative model specifications. In table S2, we show robustness to alternative fixed-effect specifications, including the inclusion of a subexperiment-by-state FE and the exclusion of year-by-subexperiment FEs. The model results are not especially sensitive to these variations. In table S3, we adopt an alternative logic for assigning geography that uses geocoded internet protocol (IP) addresses to determine workplace locations when other sources of information are missing. Here, we find a similar pattern of results, but somewhat smaller effect sizes, as expected due to the measurement error that the IP addresses introduce (e.g., people sometimes take the survey at home and not at work or use virtual private networks). In table S4, we limit the sample to hourly workers who do not have managerial roles and find that the results are not sensitive to that exclusion.

### Effects of FWW laws on channels of adjustment

**Fig. 2.**
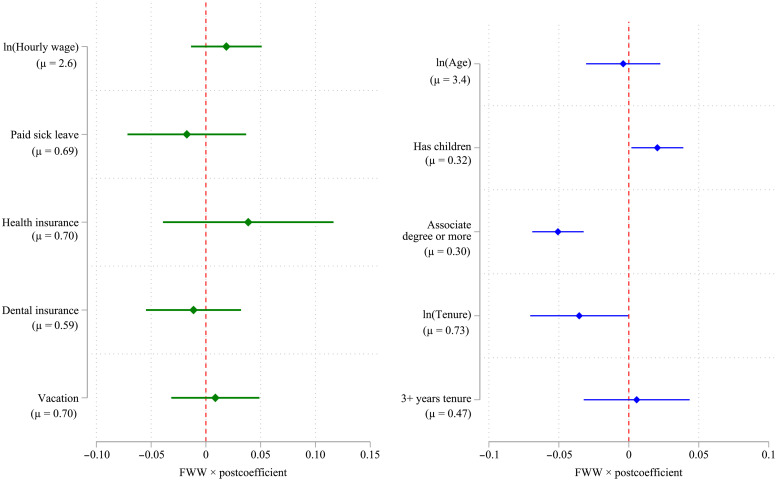
Channels of adjustment and compositional changes in response to FWW laws. SEs clustered at geographic level. All models include year × subexperiment FEs, employer × subexperiment FEs, individual-level controls, and area-level controls. *N* = 285,993. Pretreatment treatment group mean in parentheses.

Table 1 provides evidence that FWW laws led to some of the intended improvements in work schedule conditions, in particular increasing schedule notice and reducing exposure to clopening shifts and last-minute timing changes. In Fig. 2, we examine whether these laws had effects on potential channels of adjustment, such as wages and fringe benefits or on worker composition. If FWW laws exert downward pressure on wages and benefits, then these unintended effects could undercut any benefits of improved schedules from the perspective of covered workers. If FWW laws changed the composition of workers, then that could suggest one pathway through which employers adjust to these new mandates.

Overall, we see no evidence that employers reduced wages or benefits in response to new FWW schedule requirements or of sizeable shifts in the composition of workers. The left panel of Fig. 2 shows that those covered by FWW regulations experienced similar evolution of their hourly wages compared with their counterparts who were not covered by FWW laws. We also do not see any evidence that those newly covered by FWW laws experienced declines in any of a set of fringe benefits including paid sick leave, employer-sponsored health and dental insurance, or paid vacation benefits. If anything, there is evidence of modest increases in health insurance coverage. The right panel of Fig. 2 shows limited evidence of changes in the composition of workers. We find no effects on age or long job tenure but evidence of modest reductions in average tenure, reductions in the share of workers with at least some college education, and modest increases in the share of workers with children. At a minimum, we can reject that the FWW laws had any strong effects on increasing the share of workers with characteristics that might proxy for greater productivity, such as through retention or changes in the composition of applicants or new hires.

### Heterogeneity in FWW effectiveness across jurisdictions

Our main effects reveal significant but limited impacts of the FWW laws on work schedule stability and predictability and no effects on access to work hours. Prior research has called for more attention to the potential for heterogeneous treatment effects (*27*). In our case, we examine a homogenous set of firms and laws with largely similar provisions, but we anticipate that heterogeneous treatment effects could arise because of variation across FWW jurisdictions in the organizational capacity of local enforcement agencies (*25*).

**Fig. 3.**
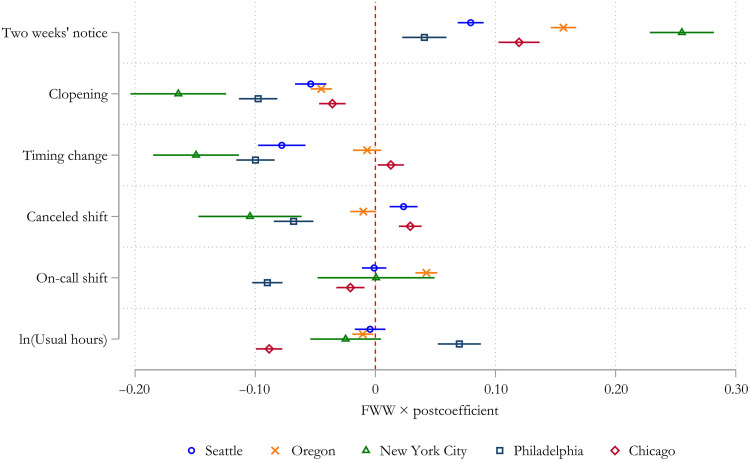
Between-jurisdiction heterogeneity in the effects of FWW laws on scheduling and work hours. SEs clustered at geographic level. All models include year FEs, employer FEs, individual-level controls, and area-level controls. *N* per subexperiment ranges from 17,032 (New York City) to 76,313 (Philadelphia). Total *N* = 285,993.

Figure 3 shows the estimated effects from jurisdiction-specific DiD models on each of the scheduling and hour outcomes. Most broadly, these results accord with a long line of research pointing to potential treatment effect heterogeneity (*41*). Across all five jurisdictions, FWW laws increased the share of workers receiving at least 2 weeks’ notice of their work schedules and reduced the share exposed to clopening shifts, but the magnitude of these effects varied by place. We found an average effect of a 13 p.p. increase in the share of workers getting at least 2 weeks’ advance notice of their schedules (Table 1), and this effect varied from about 5 p.p. in Philadelphia to 25 p.p. in New York City. Overall, FWW laws reduced clopening shifts by 8 p.p., and this effect varied from −15 p.p. in NYC to −2 p.p. in Chicago. Notably, the largest effects were found in New York City for these two outcomes and for last-minute timing changes and canceled shifts. Although we lack direct evidence, this pattern is suggestive that New York City’s strong labor enforcement capacity and the high-profile and large penalties imposed for FWW violations (*29*, *42*) led to greater compliance with the law.

Also noteworthy is the greater consistency across locations in the sign and significance of treatment effects on 2 weeks’ notice and clopening shifts compared with other outcomes. This pattern is consistent with implementation research that found managers were often uncertain about when shift timing changes were permissible without extra pay (*40*) or exploited ambiguity in the rules to avoid compliance (*34*).

Figure 3 reveals a few instances in which effects were opposite in sign to the intended effects of the FWW laws. For instance, in Chicago, covered workers experienced a small increase in timing changes and canceled shifts and a small reduction in weekly work hours. We note that the implementation of the Chicago law took place in the turbulent early pandemic period. Although Chicago business’s requests to delay implementation were denied, the city did delay implementing some of the enforcement mechanisms (*43*).

## DISCUSSION

Work time is a fundamental aspect of job quality and has been at the forefront of labor policymaking for well over a century. Over the past few decades many industries have shifted from a 9-to-5 Monday-to-Friday work week to a 24/7 business model, and employers increasingly demand open availability from workers. These always-on expectations, coupled with low wages, appear to have a range of harmful consequences for worker health and wellbeing. Policymaking at the federal level has not kept pace with these labor market shifts, but a handful of large cities and one US state have taken action to regulate work schedule conditions through a recent wave of FWW legislation. These new laws have been targeted at retail and food service industries in which workers are paid by the hour and schedule unpredictability and instability are widespread. Here, we provide the most comprehensive examination to date of whether and how these new regulations affected work scheduling conditions and other aspects of job quality and how their effectiveness differed across jurisdictions.

We draw on original data that we collected over the course of 7 years and 14 repeated cross-sectional survey waves from more than 87,000 hourly workers in the service sector across the United States. We harness recent methodological innovations to inform our stacked DiD modeling approach. This approach allows us to estimate the effects of a set of FWW laws that shares common provisions but was implemented at different time points between 2017 and 2021 in Oregon, Seattle, New York City, Philadelphia, and Chicago. These data and methods are uniquely well suited to provide a comprehensive assessment of the effects of a new wave of FWW labor policymaking.

We find that FWW laws had some, but not all, of their intended effects on work schedule conditions for those employed in the service sector. The strongest and most robust effects of these laws are on increasing the share of workers who receive at least 2 weeks of advanced notice of their work schedules. We estimate that these laws increase the share of workers receiving at least 2 weeks’ advance notice by 13 p.p., about a 29% increase. These results are in line with earlier findings from an examination of the Secure Scheduling Ordinance in Seattle (*32*). An evaluation of the Seattle FWW law demonstrated how resource intensive it was to educate employers and workers on the new regulations and to pursue the complaint-based enforcement of violations (*44*). Therefore, it was an open question whether the FWW laws would be as successful in increasing schedule notice in other locations. Our results suggest that these laws have been broadly successful in moving the needle on schedule notice. This aligns with implementation research showing that the 2 weeks’ advance notice provision was the most straightforward for employers to implement (*20*).

Among the different indicators of schedule stability, the effects on advance notice were the largest in magnitude, but the FWW laws also led to an 8-p.p. reduction in the share of workers who worked back-to-back closing and then opening shifts without adequate time for rest in between and a 6-p.p. reduction in exposure to last-minute shift timing changes. In contrast, we find no evidence that FWW laws reduced shift cancelations or on-call shifts or increased access to more work hours for involuntary part-time workers. Although these laws did improve schedules, they did not do so on all dimensions of schedules they aimed to improve.

Improvements in schedules could lead to downward pressure on other aspects of job quality. If employers experience increased labor costs because of the predictability pay requirement for instance, then they might then be less likely to increase wages or might cut back on fringe benefits. To provide a fuller accounting of the effects of these local labor laws, we also investigated the possibility that improvements in schedules may have led to countervailing retrenchment in other aspects of job quality such as wages and fringe benefits. We find no evidence of such retrenchment, nor do we find evidence of re-allocation of workers in response to the law, with stability in key worker demographics.

The implementation of FWW laws and enforcement of their provisions require substantial local resources and organizational capacity to educate workers and employers about new rights and responsibilities, investigate complaints, and penalize violations. Although our analysis examines FWW laws that are fairly uniform in their provisions and focuses on a common set of large covered firms, the resources and organizational capacities for implementation and enforcement are not uniform across jurisdictions (*25*). Given this variation, it is not unexpected that the effectiveness of the FWW laws varied across locations. Our examination of heterogeneous treatment effects revealed that the covered workers in New York City experienced the largest improvements in their schedule conditions. Notably, New York City is also the location with the most high-profile enforcement actions (*29*, *30*). Our results then reinforce the idea that the degree to which FWW laws can move the needle on scheduling for workers is contingent on local capacity to implement and enforce the laws (*25*). A valuable direction for future research would be to detail organizational capacity and practice across local enforcement agencies to refine understandings of best practice around enforcement of FWW laws and to conduct research estimating the effects of specific enforcement strategies such as high-profile publicly announced settlements and public information campaigns.

This work is subject to some important limitations. While the Shift Project data provide rare over-time measurement of scheduling and worker outcomes for a policy-relevant target population, the data are not based on a probability sample. Although prior work finds that the data accurately capture the characteristics of the target population and key associations (*45*), the potential for bias remains. It is also possible that implementation of the laws improved over time, but the relatively small sample sizes in our data prevent us from reliably estimating event-study models that might reveal such phase-in effects (though see fig. S3). In addition, we are limited to measuring the occurrence of clopening, canceled shifts, timing changes, and on-call scheduling in the prior month but lack measurement of the intensity of such exposures, which could also be shifting. Last, because our sample conditions on employment, we do not estimate the effects of FWW laws on employment levels. However, we note that Pickens and Sojourner’s (*36*) recent estimates of null effects on employment in NYC are all the more notable given that we estimate some of the largest effects on schedule stability in NYC.

In sum, we provide the most comprehensive evidence to date on the effects of FWW laws on work schedule stability and predictability, account for potential unintended consequences through channels of adjustment, and show that, in light of notable between-jurisdiction heterogeneity, the ceiling on effects is considerably higher than implied by the average across places.

## MATERIALS AND METHODS

### Research design

Prior research on FWW laws has been constrained by a lack of appropriate data, including (i) measurement of scheduling, channels of adjustment, and worker outcomes; (ii) sufficient information to identify covered workers by both employer size (essentially large-chain employers in food service and retail) and location of workplace; and (iii) breadth both in terms of geography and time to cover laws across the country that went into effect over approximately a decade.

Governmental administrative datasets such as the Longitudinal Employer-Household Dynamics (LEHD) data are large scale and could potentially identify covered workers but do not contain any detailed measures of work scheduling. Private administrative data, such as from scheduling software vendors or individual employers, have the virtue of detailed measurement of scheduling, information to gage coverage, and breadth to cover various jurisdictions, but these data are in practice almost never made available to researchers (especially to researchers whose estimates might find evidence of widespread noncompliance) and further lack information about downstream outcomes. Very-large nationally representative survey datasets, such as the CPS and American Community Survey (ACS) lack measurement of work schedules. Smaller longitudinal datasets such as the National Longitudinal Survey of Youth (1997) (NLSY-97), the Panel Survey of Income Dynamics (PSID), and the General Social Survey (GSS) have at times recently included measurement of work scheduling outcomes alongside other dimensions of job quality and worker outcomes, but in practice lack sufficient sample size and identifying information to capture covered workers in treated locations.

We sought to fill this data gap by fielding an original repeated cross-sectional survey targeted to exactly the workers covered by FWW laws that included detailed measures tailored to the task of evaluation. To do so, we developed a method to target workers at a large set of specific covered firms in retail and food service at a national scale and invite them to complete a focused survey. While there is no national sampling frame that includes workers’ employers, Meta’s advertising platform contains detailed information about multiple user characteristics that can be used to create “audiences” of Facebook and Instagram users to deliver targeted advertisements (this is the basis of Facebook’s $130 billion revenue advertising business). We use this tool to create firm-specific audiences/sampling frames and then contact these potential respondents to invite them to complete a survey using paid advertisements on those same platforms. These survey recruitment advertisements then appeared in users’ feeds, inviting them to click-through to a Qualtrics survey, consent, and participate.

On the continuum from gold-standard probability sample surveys that have a complete sampling frame and very-high response rates (essentially government surveys such as the CPS with a response rate of 70%) to opt-in nonprobability online panels (such as those maintained by firms such as Cint or Prolific), our approach occupies a middle ground. There is certainly scope for bias in terms of selection into Facebook/Instagram use (although it remains very high, at about 80%, among working-age adults), into identification as a member of the target population on the platform (we estimate that about 41% of all workers at our target firms are so identified by comparing audience sizes, which themselves may be subject to errors in Meta’s process, to the ground-truth Reference USA database of firms), and into survey completion (∼1.1% of those who see the ad contribute survey data). However, unlike with the online panel vendors, respondents are not actively opting in to take large numbers of surveys. Furthermore, our data appear to closely align with gold-standard benchmarks such as the CPS and NLSY-97 on both univariate statistics such as wages and scheduling and on bivariate associations, such as the wage returns to tenure (*45*), which tracks recent work suggesting that nonprobability samples often reproduce associations, if not levels (*46*–*48*).

We draw on data collected using this approach from hourly workers employed at one of 217 large retail and food service firms. These data were collected in 14 twice-annual repeated cross sections between March of 2017 and November of 2023. We limit the sample to respondents with complete data on all model covariates (below) for a final analysis sample of 87,123 respondents. We describe the sample characteristics in table S5.

### Key measures

#### FWW exposure

Treated workers are defined as those employed in a workplace covered by the relevant FWW ordinance. There are two components to assigning coverage—whether the firm is a covered firm and whether the workers’ establishment is within a covered jurisdiction. For the former, all of the firms at which respondents work are sufficiently large in terms of workers and establishments so as to be covered by the FWW ordinances. The Seattle, Oregon, Chicago, and Philadelphia laws cover both retail and food service worker, and so workers at the full set of firms are included. New York City’s law specifically applies to fast-food workers (a much weaker law covers retail workers, and we do not examine that law here). In table S6, we describe the dates and provisions of each of these five ordinances. We also present the details of five other ordinances that we do not study because passage was too early for us to have pretreatment data (San Francisco), because the jurisdiction was too small to allow sufficient sample size in our data (Emeryville, Berkeley, and Evanston), or passed too late to allow sufficient posttreatment observation (LA and also Berkeley and Evanston).

Figure S1 shows the full set of employers and how they are treated by subexperiment. The stacked data structure, described below, allows us to tailor our sample—both treatment and control—to these city-specific criteria, dropping workers outside of the covered industries from the analysis sample in those stacks.

For the latter group, we use a multistep procedure to assign respondents to a specific treated location or to a specific county that is not treated, which we map out in fig. S2. First, respondents are asked to report their workplace’s “number” (such as Walmart Super Center #3081), address, and/or phone number. We complement this data by scraping store finder websites for the firms in the sample and then use exact and fuzzy matching to assign a precise geographic location of the workplace that we can use to assign respondents to a treated location or a control county. In addition, at most survey waves, we directly asked respondents for the state of their workplace and then, when the state included a covered jurisdiction, whether their workplace was located within the relevant covered jurisdiction (i.e., “Is your [EMPLOYER] workplace located within Seattle city limits?”). Our main specification uses only these two data sources to assign respondents to a treated location or to control states. Using this approach, as shown in table S7, we assign 88% of the overall sample to treated location or to control and exclude 12,359 cases. Table S7 contrasts these samples in terms of demographic and workplace characteristics. The two groups are very similar, suggestive that this approach does not introduce concerning bias.

As a robustness check, the location of respondents who lacked information from both of the aforementioned data sources was determined by geo-coded IP address. We then reestimated our models using this complete sample (table S3). Our main results hold, but we believe that using IP locations adds noise to our estimates due to use of VPNs and use while commuting, among other factors. Together, these sources of workplace location information allow us to define a dichotomous variable equal to “1” if the respondent is at a covered employer/location and “0” if otherwise.

Last, we define a dichotomous variable that is equal to 0 if the survey observation was collected before the law going into effect and 1 if it was collected after the law went into effect. This time variable is, necessarily, defined in terms of time relative to the city-specific events because the laws went into effect at different times. The stacked data structure, described below, accommodates this coding.

#### Work schedule stability

We measure six worker scheduling outcomes related to the FWW law provisions. First, following Harknett *et al.* (*32*), we define a dichotomous variable equal to 1 if workers report receiving at least 2 weeks’ notice of their schedule and 0 if they receive their schedules with less notice. This closely tracks perhaps the key provision of FWW laws requiring 2 weeks’ notice. Second, we coded workers as 1 if they report working a “clopening shift” or “worked a closing shift and then worked the very next opening shift with less than 11 hours off in between your shifts” in the last month and zero otherwise. Third, we code workers as 1 if they reported having a shift canceled in the last month and 0 otherwise. Fourth, we construct a measure of on-call shifts equal to 1 if workers reported being asked to “be on-call, available to work, and you find out if you are needed to work just a few hours before your shift” in the last month and 0 otherwise. Fifth, we create a similar measure, capturing if, in the last month, “your employer ever changed the timing or the length of your scheduled shift. For example, your employer asked you to come in early or late or asked you to leave early or to stay later than the hours you were originally scheduled for.” Last, workers report on their usual weekly work hours, which we treat as a continuous variable.

### Channels of adjustment

We gauge response through such channels of adjustment using (i) self-reported hourly wage and workers’ direct reports of access to (ii) paid sick leave, (iii) employer sponsored health insurance, (iv) employer sponsored dental insurance, and (v) paid vacation time.

### Individual-level controls

Compared with administrative data from work scheduling software, a key benefit of our data is that we observed a rich set of individual-level characteristics that could confound our key estimates. We control then for race/ethnicity, age, gender, marital status, parenthood, school enrollment, speaking a language other than English at home, tenure at current job, union membership, and managerial status.

### Area-level controls

We also construct and adjust for a large set of area-level characteristics that could similarly confound our key estimates. We draw on data from the Bureau of Labor Statistics to measure county-month level unemployment and on data from the Union Membership and Coverage Database (derived from CPS) to capture state-year level unionization (*49*, *50*). We use US Census Bureau population estimates to measure county-year population size, percent female, percent white non-Hispanic, percent Black, non-Hispanic, percent Hispanic, and percent in age groups by decade of life lived (*51*, *52*). We draw on state and local government websites and the Shift Project’s Labor Policy Database to create area-level estimates (state and local levels) of whether there is a minimum wage standard in excess of the federal standard in effect and whether there is a paid sick leave policy in effect and/or a paid family and medical leave policy in effect (*53*).

### Statistical analysis

DiD methods are particularly useful tools when estimating causal effects in situations in which random experimental manipulations are not possible. In its most simple version, a DiD approach includes two time periods, one before treatment (pre) and one after treatment (post), and two groups, one that was never treated and one that was treated starting in the post period. However, real-life nonexperimental scenarios seldom are that simple. They often include multiple groups that received treatment at different times, were not observed for the same amount of time before and after treatment, and do not have an equal number of observations across the different time periods. We face all of those issues and thus turn to recent methodological work that advances a set of responsive empirical practices.

Until a few years ago, many researchers who encountered DiD settings in which units are treated at different times used two-way fixed effects (TWFE)—i.e., time and group FEs—to account for the variation introduced by the different treatment times (*54*). However, numerous researchers in the last few years have shown that DiD approaches with TWFE regressions do not produce a straightforward and intuitive estimate but rather include implicit weights that obscure the interpretation of the coefficients (*55*–*57*). Multiple authors have proposed different solutions to this and other adjacent problems in DiD approaches (*55*, *57*–*60*). Among these, we follow and adapt Wing *et al.* (*60*) stacked DiD method due to its adaptability to our case and its intuitive explanation.

The stacked DiD method builds on the intuitive estimator of the simple 2 × 2 DiD case. Wing *et al.* (*60*, *61*) general idea is that rather than trying to fit a TWFE model to the whole sample, a more straightforward approach would be something akin to running individual subexperiments that resemble the well-understood and simple 2 × 2 DiD case (two groups, two periods, and all treated units treated at the same time) and then taking an average of the estimated treatment effects corresponding to each of these simple 2 × 2 DiD models. They suggest doing so by assembling a stack of simple 2 × 2 DiD cases, which they call subexperiments, and then running a weighted ordinary least squares (OLS) model without FEs on the stack. This setting allows the researchers to tailor each smaller 2 × 2 subexperiment to the requirements of the particular case (e.g. different covered industries, as is our case) and have a clear understanding of the comparison groups and periods.

In our case, we build the stack by defining a different subexperiment per treated location (Seattle, Chicago, New York City, Philadelphia, and Oregon). The treatment group for a particular subexperiment consists of those observations that are treated at the corresponding location within the observed time frame, while the control group includes those in other locations that will never adopt treatment. Thus, control observations in, for example, Boston appear once in multiple subexperiments, but treatment observations only appear in the subexperiment corresponding to their location. Each subexperiment has its own event time, and thus, the pre and post periods are defined differently for each subexperiment according to the time at which the location was treated. Consequently, a control observation can appear in the pre period in Philadelphia, while it might appear in the post period in Oregon. We restrict the sample to food service workers in the New York City subexperiment. After defining the relevant treatment and control groups for each subexperiment, the datasets for all subexperiments are row bound or vertically “stacked” into a large dataset.

We construct the stacks so that each subexperiment/jurisdiction has exactly six posttreatment periods. Trimming the post period in this way minimizes the risk of bias from dynamic or heterogeneous treatment effects that could unfold across the subexperiments and skew the overall post-period average effect. We are more limited in our ability to completely harmonize the length of the pre-period observation window across the subexperiments. The earliest Shift Project data that we can use are from spring of 2017. That allows us to have a full six pre periods for Chicago and Philadelphia. However, it limits us to three pre-period waves in Oregon, two pre-period waves in NYC, and one pre-period wave in Seattle. Unlike the possibility that posttreatment effects could unfold dynamically, we do not have priors that the pre periods would show this kind of dynamic variation. However, to check that the main results are not sensitive to trimming decisions, we ran models trimming to exactly one period in the pre period and six in the post period—which we have for all jurisdictions included in the paper—and our estimates show a qualitatively similar set of results although less precisely estimated.

In table S8, we use this stacked dataset to present a simple set of differences in means between the treatment and control group, each pretreatment versus posttreatment and the overall DiD for each outcome. We see evidence that is entirely consistent with our preferred regression-based estimates (shown in Table 1). These simple differences suggest a 15-p.p. increase in the share of workers receiving 2 weeks’ advance notice (versus 13 p.p. in our preferred models), a 10 p.p. reduction in clopening shifts (versus 8.4 p.p. in our preferred models), a 7 p.p. reduction in timing changes (versus 6.7 p.p. in our preferred models), and small differences in the other outcomes (in line with our preferred models). In table S9, we do the same exercise but by jurisdiction and again recover simple differences in means that are very consistent with our by-jurisdiction estimates in Fig. 3.

Once the stack is assembled, we run a weighted OLS regression with the scheduling measures as outcomes (we use a linear probability model rather than logit to simplify interpretation and model comparison), the interaction between treatment group indicator and period indicator (pre or post) as the main coefficient of interest, a treatment group indicator, a period group indicator, and the individual and area-level controls described above. Because we are pooling observations from different time points into one pre and one post period, our preferred specification includes year-by-subexperiment FEs. We also add employer-by-subexperiment FEs to absorb potential changes in sample composition by employer and to better capture the effect of the laws on employer behavior. The ability to include employer FEs is a valuable and unusual tool allowed for by the Shift data, aligning the comparisons not just to untreated workers in the same industries but making for within employer comparisons. In robustness, we also test the inclusion of state-by-subexperiment FEs.

The reason why a weighted OLS regression is necessary is that, to correctly identify a straightforward and causal estimate with the stacked data, we need to account for the fact that, due to the construction of the stack, the control and treatment groups are not weighted equally. We resolve this issue by following the general weighting strategy outlined in Wing *et al.* (*60*). We construct weights to produce an average causal effect on the treated that weighs each subexperiment equally, regardless of the sample size of each subexperiment. We also test robustness to weighting subexperiments proportionately to number of treated units and to not weighting.

Our approach differs from Wing *et al.* (*60*, *61*) in that we use individual-level data in the stack, whereas Wing *et al.* (*60*, *61*) aggregate individual-level observations into treated-group–level observations before assembling the stack and fitting the models. Given that our data are repeated cross sections with variable sample sizes, using individual-level observations means that we have different sample sizes for the control and treated units in the pre and post periods. The weights described in Wing *et al.* (*60*, *61*) assume equal number of observations in the pre and post periods by treatment group and subexperiment, so we had to construct weights separately for pre and post observations following the same principle that Wing *et al.* (*60*, *61*) use to calculate the weights. Similarly, Wing *et al.* (*60*, *61*) suggest clustering SEs at the observation unit or unit-by-subexperiment level. Instead, we cluster our SEs at the geographic location level, defined as city level for those cities that were ever treated and state level for all observations not in those cities.

We also ran models using a coarsened exact matching (CEM) approach to constructing control groups. In these models, we matched most treatment observations to control observations in the corresponding pre or post period using race, gender, highest degree attained, parenthood status, and categorical coarsened versions of the linear age and work tenure variables used elsewhere. We fitted these CEM models with each of the weighting approaches mentioned above—no weights, equal weight by location, and weight by proportion of treated units—and the results maintained their general shape.

Separately, we produce similar estimates for each treated jurisdiction. This approach allows us to examine how the effect at each location is contributing to the estimate of the overall effect. In the models about only one location, we essentially run a simple 2 × 2 DiD in which all treated units were treated at the same time and, consequently, event and calendar time are the same. It is equivalent to examining each of the subexperiments in the stack individually. We include the same controls as in the stacked case, as well as year and employer FEs. The only difference is that the weights become unnecessary, as there is no stacked DiD to account for.

While the Wing *et al.* (*60*, *61*) method has the advantage of allowing us to tailor the control group to the jurisdiction, we also assess the sensitivity of our results to an alternative estimator, that of Callaway and Sant’Anna (*55*). We use the repeated cross-sectional version of the estimator, using regular OLS (linear probability models) in the models, and we include the same individual-level and area-level controls as in our original specification, except for the year-by-subexperiment FEs that are rendered superfluous by the Callaway and Sant’Anna (*55*) approach. We estimate the Average Treatment Effect on the Treated (ATT) for each location and time and aggregate all the post and pre times, as well as all the jurisdictions as per the Callaway and Sant’Anna (*55*) aggregation procedure, using equal weights per jurisdiction.

In table S10, we show the results of this analysis. First, in M1, we show our preferred estimates from the Wing *et al.* (*60*, *61*) models, reproducing the results in M3 of Table 1. As noted above, the advantage of this model is that it allows us to tailor the comparison group for NYC’s FWW law to other fact food workers. In M2 of table S10, we reestimate this preferred model but include retail workers in the NYC comparison group. This approximates the uniform comparison group that is required in the Callaway and Sant’anna (*55*) approach. As we would expect, we find some attenuation of effects. The effect of 2 weeks’ notice goes from 0.130 in M1 to 0.091 in M2, and the reductions in clopening and timing change are also somewhat smaller. We see entirely consistent estimates in the null effects on canceled shifts, on-call shifts, and usual hours. In M3, we show the estimates from using the Callaway and Sant’anna (*55*) approach. Here, we find consistent effects of the laws on 2 weeks’ notice of schedules, B = 0.120, and on clopening, B = −0.050 (but is not significant). We also find consistent null effects on canceled shifts and usual hours. However, the effect on timing changes is not present in M3, and there is a marginally significant increase in on-call shifts not found in the other models.
