# Supplementary material for: Fair Workweek laws in the US: An appraisal of intended and unintended consequences
Source: Sci Adv. 2026 Jun 24;12(26):eaea8632. doi: 10.1126/sciadv.aea8632 (PMC13292935; doi:10.1126/sciadv.aea8632)
Supplement: Supplementary file 1 — Figs. S1 to S3 Tables S1 to S10 [file sciadv.aea8632_sm.pdf]

Science Advances
AAAS

Supplementary Materials for

Fair Workweek laws in the US: An appraisal of intended and unintended consequences

Daniel Schneider *et al.*

Corresponding author: Daniel Schneider, dschneider@hks.harvard.edu

Sci. Adv. **12**, eaea8632 (2026)
DOI: 10.1126/sciadv.aea8632

This PDF file includes:

Figs. S1 to S3
Tables S1 to S10

7-Eleven	Carl's Jr.	Gap	Kroger/QFC	PetSmart	Staples
99 Cents Only Stores	Checker's Drive-In	Giant	La Quinta	Pizza Hut	Starbucks
Abercombie & Fitch	Cheesecake Factory	Giant Eagle	Levi's	Popeye's	Steak 'n Shake
Ace Hardware	Chick-Fil-A	Golden Corral	Little Caesars	Potbelly	Stop & Shop
Acme Markets	Chili's	H&M	LongHorn Steakhouse	Price Chopper	Subway
Advance Auto Parts	Chipotle	Hannaford	Lowe's	Publix	T.J. Maxx
Albertsons	Church's Chicken	Harbor Freight Tools	Macy's	Qdoba	Taco Bell
Aldi	Cold Stone Creamery	Hardee's	Maggiano's	QuikTrip	Target
Allegiance/Foodtown	Costco	Harris Teeter	Marriott	QVC	Texas Roadhouse
Amazon	Cracker Barrel	HEB	Marshalls	Rally's	TGI Friday's
American Eagle	Crowne Plaza Hotels	Hilton	McDonald's	Ralph's	The Fresh Market
Apple	Culvers	Hobby Lobby	Meijer	Red Lobster	Tim Horton's
Applebees	CVS	Holiday Inn	Menards	Red Robin	T-Mobile
Arby's	Dairy Queen	Home Depot	Michaels	REI	Tractor Supply Co.
AT&T	Days Inn	HomeGoods	Mobil	Rite Aid	Trader Joe's
Athleta	Denny's	Hyatt	Moe's Southwest Grill	Ritz Carlton	Ulta Beauty
Au Bon Pain	DHL	Hy-Vee	Nike	Ross	UPS
AutoZone	Dick's Sporting Goods	IHG	Noodles & Co.	Round Table	Urban Outfitters
Babies 'R' Us	Dillard's	IHOP	Nordstrom	Ruby Tuesday	Verizon
Banana Republic	Disney	Ikea	Office Depot	Safeway	Victoria's Secret
Barnes & Noble	Dollar General	In-N-Out Burgers	OfficeMax	Saks Fifth Avenue	Vons
Bartell Drug	Dollar Tree	J.Crew	Old Navy	Sams Club	Waffle House
Bath & Body Works	Domino's	Jack in the Box	Olive Garden	Sears	Walgreens
Bed Bath & Beyond	Dunkin Donuts	Jamba Juice	O'Reilly Auto Parts	Sephora	Walmart
Best Buy	Express	Jason's Deli	Other	Shaw's	Wawa
Best Western	Exxon	JCPenney	Outback Steakhouse	Shell	Wegmans
Big Lots	Family Dollar	Jersey Mike's Subs	P.F. Chang's	Sheraton	Wendy's
BJ's Restaurants	Fedex	Jiffy Lube	Panda Express	Sherwin Williams	Westin
BJ's Wholesale	Five Guys	Jimmy John's	Panera	ShopRite	Whataburrger
Bob Evans	Food 4 Less	Jo-Ann Fabrics	Papa John's	Smashburger	White Castle
Bojangles	Food Lion	KFC	Papa Murphy's	Smith's Food and Drug	Whole Foods
Boscov's	Food Locker	Kimpton Hotels	Payless Shoes	Sonic	Wingstop
Buffalo Wild Wings	Forever 21	Kmart	PCC Natural Markets	Speedway	Winn Dixie
Burger King	Fred Meyer	Kohls	Perkins	Sprint	Wyndham
Burlington	GameStop	Krispy Kreme	Petco	Sprouts Farmers Market	XPO Logistics
					Zaxby's

Retail: not treated in NYC
Fast food
Other food service: not treated in NYC
Hospitality: not treated in SEA or NYC

Total: 211 firms

Fig. S1. Assignment of Employers to Treatment by Jurisdiction.

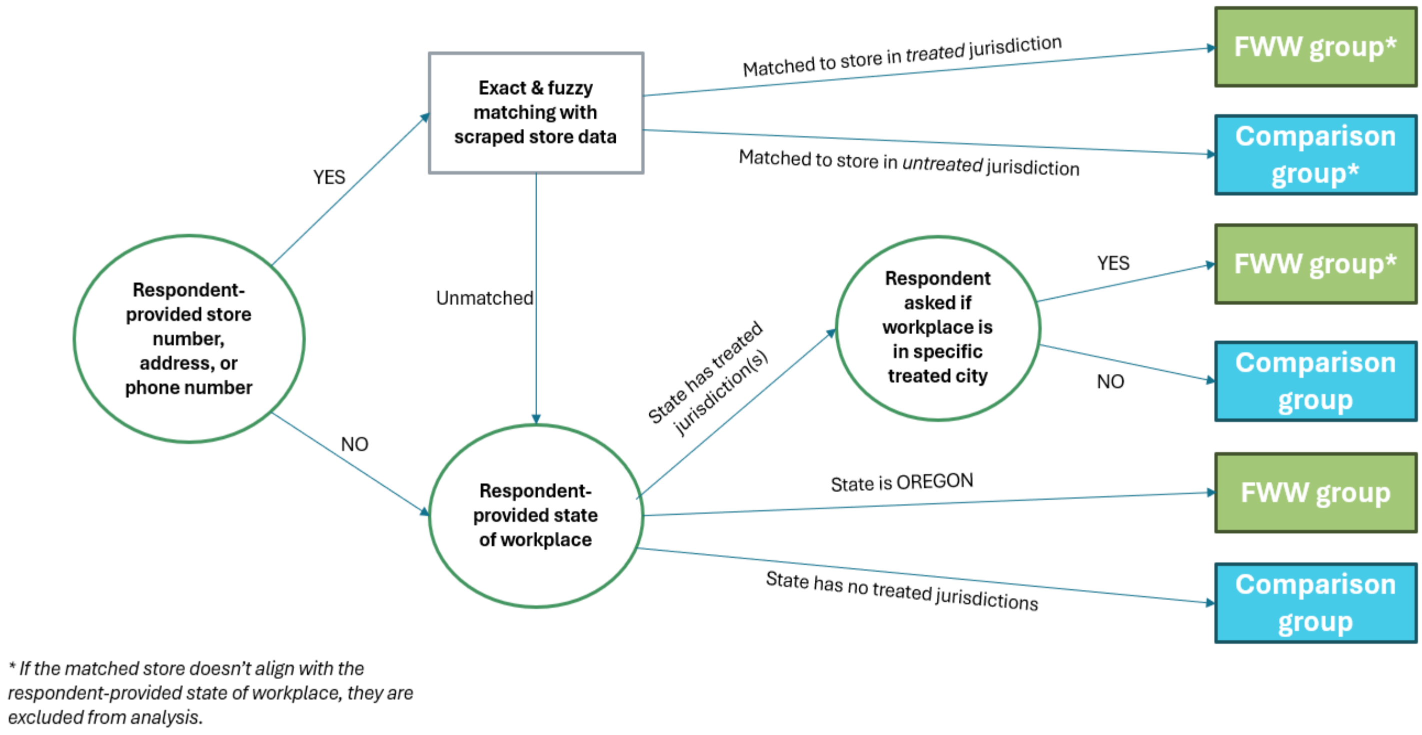

Fig. S2. Process of Geographic Assignment of Respondents.

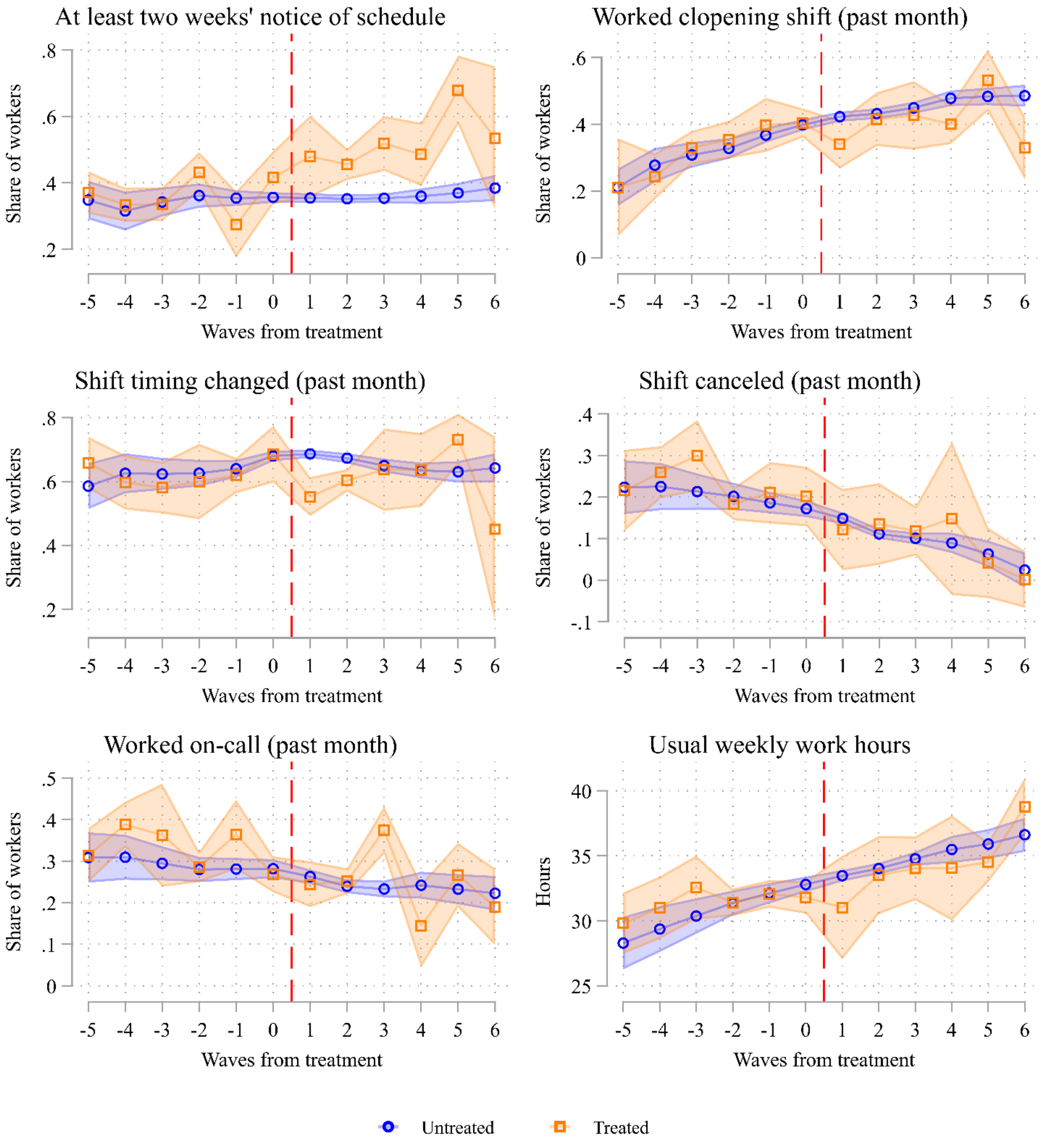

Fig. S3. Event Study Plots of Effects of Fair Work Week Laws on Scheduling and Work Hours. Weighted using equal sub-experiment weights. Standard errors clustered at geographic level. Includes year × sub-experiment fixed effects, employer × sub-experiment fixed effects, individual-level controls, and area-level controls. 0 = last pre-treatment period, 1 = first post-treatment period. Shaded area represents 95% confidence intervals. N = 285,993.

	Non-CEM			CEM		
	(1)	(2)	(3)	(4)	(5)	(6)
Two weeks' notice						
FWW × Post	0.130***	0.136***	0.144***	0.135***	0.133***	0.138***
	(0.032)	(0.020)	(0.019)	(0.041)	(0.025)	(0.024)
Clopening						
FWW × Post	-0.084***	-0.055***	-0.055***	-0.084***	-0.055***	-0.059***
	(0.025)	(0.010)	(0.012)	(0.02)	(0.008)	(0.013)
Timing change						
FWW × Post	-0.067*	-0.032	-0.021	-0.071*	-0.033+	-0.027
	(0.029)	(0.020)	(0.016)	(0.034)	(0.017)	(0.017)
Canceled shift						
FWW × Post	-0.026	-0.007	-0.005	-0.031	-0.009	-0.004
	(0.021)	(0.010)	(0.013)	(0.026)	(0.011)	(0.014)
On-call shift						
FWW × Post	-0.013	0.019	0.020	-0.020	0.017	0.019
	(0.019)	(0.017)	(0.015)	(0.022)	(0.018)	(0.016)
Usual hours						
FWW × Post	-0.373	-0.305	-0.401+	-0.264	-0.268	-0.271
	(0.723)	(0.259)	(0.223)	(0.712)	(0.270)	(0.197)
N	285993	285993	285993	167775	167775	167775
Avg. sub-experiment *N*	57199	57199	57199	33555	33555	33555
Year × sub-experiment FE	X	X	X	X	X	X
Employer × sub-experiment FE	X	X	X	X	X	X
Individual-level controls	X	X	X	X	X	X
Area-level controls	X	X	X	X	X	X
Equal sub-experiment wts.	X			X		
Treatment unit share wts.		X			X	

Table S1. Robustness of Main Effects of Fair Work Week Laws to Alternative Matching and Weighting. + $p < 0.10$, * $p < 0.05$, ** $p < 0.01$, *** $p < 0.001$. Standard errors clustered at geographic level. CEM (models 4-6): coarsened exact matching, on basis of demographic and work characteristics. Three different weighting specifications: sub-experiments weighted equally (models 1 & 4), sub-experiments weighted proportionately to the number of treated units (models 2 & 5), and unweighted (models 3 & 6).

	(1)	(2)	(3)
Two weeks' notice			
FWW × Post	0.130***	0.133***	0.127***
	(0.032)	(0.030)	(0.029)
Clopening			
FWW × Post	-0.084***	-0.074**	-0.081***
	(0.025)	(0.024)	(0.024)
Timing change			
FWW × Post	-0.067*	-0.061*	-0.063*
	(0.029)	(0.024)	(0.026)
Canceled shift			
FWW× Post	-0.026	-0.029	-0.030
	(0.021)	(0.020)	(0.025)
On-call shift			
FWW × Post	-0.013	-0.014	-0.016
	(0.019)	(0.020)	(0.020)
Usual hours			
FWW × Post	-0.373	-0.461	-0.458
	(0.723)	(0.740)	(0.793)
N	285993	285993	285993
Avg. sub-experiment *N*	57199	57199	57199
Individual-level controls	X	X	X
Area-level controls	X	X	X
Employer × sub-experiment FE	X	X	X
Year × sub-experiment FE	X		X
State × sub-experiment FE			X

Table S2. Robustness of Main Effects of Fair Work Week Laws to Alternative Fixed Effects Specifications. + $p < 0.10$, * $p < 0.05$, ** $p < 0.01$, *** $p < 0.001$. Weighted using equal sub-experiment weights. Standard errors clustered at geographic level.

	(1)	(2)	(3)
Two weeks' notice			
FWW × Post	0.113***	0.111***	0.107***
	(0.020)	(0.019)	(0.020)
Clopening			
FWW × Post	-0.044***	-0.050***	-0.045***
	(0.013)	(0.006)	(0.007)
Timing change			
FWW × Post	-0.051**	-0.051**	-0.053**
	(0.019)	(0.017)	(0.018)
Canceled shift			
FWW × Post	-0.019	-0.018	-0.018
	(0.018)	(0.016)	(0.016)
On-call shift			
FWW × Post	-0.017	-0.019	-0.016
	(0.024)	(0.021)	(0.020)
Usual hours			
FWW × Post	0.382	0.208	0.313
	(0.608)	(0.686)	(0.706)
N	335880	335880	335880
Avg. sub-experiment *N*	67176	67176	67176
Year × sub-experiment FE	X	X	X
Employer × sub-experiment FE	X	X	X
Individual-level controls		X	X
Area-level controls			X

Table S3. Robustness of Main Effects of Fair Work Week Laws to Alternative Approaches to Geographic Assignment. + $p < 0.10$, * $p < 0.05$, ** $p < 0.01$, *** $p < 0.001$. Weighted using equal sub-experiment weights. Standard errors clustered at geographic level. Alternative geographic assignment: using IP address to fill in gaps in self-reported location information.

	(1)	(2)	(3)
Two weeks' notice			
FWW × Post	0.129***	0.129***	0.126***
	(0.028)	(0.027)	(0.029)
Clopening			
FWW × Post	-0.079***	-0.078***	-0.077***
	(0.022)	(0.018)	(0.020)
Timing change			
FWW × Post	-0.084**	-0.087**	-0.091**
	(0.032)	(0.030)	(0.034)
Canceled shift			
FWW × Post	-0.028	-0.029	-0.030
	(0.028)	(0.027)	(0.027)
On-call shift			
FWW × Post	-0.017	-0.017	-0.021
	(0.026)	(0.025)	(0.025)
Usual hours			
FWW × Post	-0.534	-0.234	-0.170
	(0.984)	(0.874)	(0.877)
N	214213	214213	214213
Avg. sub-experiment *N*	42843	42843	42843
Year × sub-experiment FE	X	X	X
Employer × sub-experiment FE	X	X	X
Individual-level controls		X	X
Area-level controls			X
Managers excluded	X	X	X

Table S4. Robustness of Main Effects of Fair Work Week Laws to Exclusion of Managers. + $p < 0.10$, * $p < 0.05$, ** $p < 0.01$, *** $p < 0.001$. Weighted using equal sub-experiment weights. Standard errors clustered at geographic level.

	Full Sample	Treatment	Control
**Demographics**			
Race/ethnicity			
White	0.80	0.73	0.80
Black	0.04	0.04	0.04
Hispanic	0.09	0.12	0.09
Other	0.07	0.11	0.07
Gender			
Male	0.28	0.31	0.28
Female	0.70	0.67	0.70
Age			
18-19	0.12	0.12	0.12
20-29	0.32	0.39	0.31
30-39	0.16	0.18	0.16
40-49	0.14	0.13	0.14
50-59	0.16	0.13	0.16
60+	0.10	0.06	0.10
Educational attainment			
No high school diploma	0.04	0.05	0.04
High school diploma	0.34	0.29	0.34
Some college	0.38	0.38	0.38
College degree (associate or higher)	0.24	0.28	0.24
Cohabitation status			
Married	0.29	0.24	0.29
Living with partner	0.21	0.24	0.21
Not living with partner	0.50	0.52	0.50
Has children	0.48	0.38	0.48
**Work characteristics**			
Job tenure			
Two years or less	0.49	0.52	0.49
Three to five years	0.22	0.22	0.22
Six or more years	0.28	0.26	0.28
Manager	0.25	0.22	0.25
Union member	0.08	0.18	0.07

**Geographic characteristics**			
County population	768018	1281248	742400
County unemployment rate	4.80%	4.83%	4.80%
Policy coverage			
Covered by state/local min. wage	0.61	0.94	0.59
Covered by PSL mandate	0.32	1.00	0.29
Covered by PFML mandate	0.18	0.24	0.18
County-level demographics			
Pct. female	0.51	0.51	0.51
Race/ethnicity			
White (non-Hispanic)	0.79	0.77	0.80
Black (non-Hispanic)	0.11	0.08	0.12
Hispanic	0.14	0.14	0.14
Age			
15-19	0.07	0.06	0.07
20-29	0.14	0.14	0.14
30-39	0.13	0.15	0.13
40-49	0.12	0.13	0.12
50-59	0.13	0.13	0.13
60-69	0.12	0.12	0.12
70+	0.11	0.10	0.11
State union coverage	0.12	0.17	0.11
N	87123	4142	82981

Table S5. Characteristics of Analysis Sample. Race/ethnicity categories are mutually exclusive (i.e., respondents reporting Hispanic and 1+ other race/ethnicity are classified as Hispanic).

Jurisdiction	First implemented	Fully enforced	Covered industries	Advance notice (days)	Predictability pay			Clopening		Included in analysis?
					Timing changed	*Shift canceled/ reduced*	*On-call*	*Hour buffer*	*Reqs.*	
**San Francisco, CA**	Jan. 2015	Jul. 2015	Retail, food service	14	1-4 hours pay per instance	1-4 hours pay per instance	2-4 hours pay if not called in	*N/A*		No (insufficient pre-period data)
**Seattle, WA**	Jul. 2017	Jan. 2018	Retail, food service	14	1 hour pay per instance	0.5x pay of lost hours	0.5x pay of hours not worked	10	Right to decline, 1.5x pay if accepted	Yes
**Oregon**	Aug. 2017	Jan. 2019	Retail, food service, hospitality	7	1 hour pay per instance	0.5x pay of lost hours	0.5x pay of hours not worked	10	Right to decline, 1.5x pay if accepted	Yes
	Aug. 2018			14						
**Emeryville, CA**	Jul. 2017	Jan. 2018	Retail, food service	14	1 hour pay per instance	1-4 hours pay per instance	No pay for not being called in, same pred. pay as for regular shifts	11	Right to decline, 1.5x pay if accepted	No (small treated jurisdiction)
**New York City, NY**	Nov. 2017		Retail	3	Up to $500 per instance			*N/A*		No (anomalously weak advance notice requirement)
			Fast food	14	$10-$15 bonus per instance	$20-$75 bonus per instance	No pay for not being called in, same pred. pay as for regular shifts	11	Right to decline, $100 bonus per instance if accepted	Yes

<table>
<tr><th rowspan="2">Jurisdiction</th><th rowspan="2">First implemented</th><th rowspan="2">Fully enforced</th><th rowspan="2">Covered industries</th><th rowspan="2">Advance notice (days)</th><th colspan="3">Predictability pay</th><th colspan="2">Clopening</th><th rowspan="2">Included in analysis?</th></tr>
<tr><th>Timing changed</th><th>Shift canceled/ reduced</th><th>On-call</th><th>Hour buffer</th><th>Reqs.</th></tr>
<tr><td rowspan="2">Philadelphia, PA</td><td>Apr. 2020</td><td rowspan="2">Jun. 2021</td><td rowspan="2">Retail, food service, hospitality</td><td>10</td><td rowspan="2">1 hour pay per instance</td><td rowspan="2">0.5x pay of lost hours</td><td rowspan="2">0.5x pay of hours not worked</td><td rowspan="2">9</td><td rowspan="2">Right to decline, $40 bonus per instance if accepted</td><td rowspan="2">Yes</td></tr>
<tr><td>Jan. 2021</td><td>14</td></tr>
<tr><td rowspan="2">Chicago, IL</td><td>Jul. 2020</td><td rowspan="2">Jan. 2021</td><td rowspan="2">Retail, food service, healthcare, hospitality, warehouse, construction, manufacturing</td><td>10</td><td rowspan="2">1 hour pay per instance</td><td rowspan="2">1 hour pay per instance, or 0.5x pay of lost hours if <24 hours' notice</td><td rowspan="2">No pay for not being called in, same predictability pay as for regular shifts</td><td rowspan="2">10</td><td rowspan="2">Right to decline, 1.25x pay if accepted</td><td rowspan="2">Yes</td></tr>
<tr><td>Jul. 2022</td><td>14</td></tr>
<tr><td>Los Angeles, CA</td><td>Apr. 2023</td><td>Sep. 2023</td><td>Retail</td><td>14</td><td>1 hour pay per instance</td><td>0.5x pay of lost hours</td><td>0.5x pay of hours not worked</td><td>10</td><td>Right to decline, 1.5x pay if accepted</td><td>No (insufficient post-period data)</td></tr>
<tr><td>Evanston, IL</td><td>Sep. 2023</td><td>Jan. 2024</td><td>Retail, food service, hospitality, warehouse, construction, manufacturing</td><td>14</td><td>1 hour pay per instance</td><td>1-4 hours pay per instance</td><td>Up to 4 hours pay per shift not worked</td><td>11</td><td>Right to decline, 1.5x pay if accepted</td><td>No (insufficient post-period data)</td></tr>
<tr><td>Berkeley, CA</td><td colspan="2">Jan. 2024</td><td>Retail, food service, healthcare, hospitality, warehouse, construction, manufacturing</td><td>14</td><td>1 hour pay per instance</td><td>1-4 hours pay per instance</td><td>No pay for not being called in, same predictability pay as for regular shifts</td><td>11</td><td>Right to decline, 1.5x pay if accepted</td><td>No (insufficient post-period data)</td></tr>
</table>

Table S6. Fair Work Week Law Provisions, by Jurisdiction.

	Main (not using IP)	Alternative (using IP)	*Excluded from main sample*
**Demographics**			
Race			
White	0.80	0.80	*0.77*
Black	0.04	0.04	*0.04*
Hispanic	0.09	0.10	*0.11*
Other	0.07	0.07	*0.08*
Gender			
Male	0.28	0.28	*0.29*
Female	0.70	0.70	*0.70*
Age			
18-19	0.12	0.13	*0.14*
20-29	0.32	0.33	*0.38*
30-39	0.16	0.16	*0.17*
40-49	0.14	0.14	*0.13*
50-59	0.16	0.16	*0.13*
60+	0.10	0.10	*0.05*
Educational attainment			
No high school diploma	0.04	0.04	*0.05*
High school diploma	0.34	0.34	*0.33*
Some college	0.38	0.38	*0.39*
College degree (associate or higher)	0.24	0.24	*0.24*
Cohabitation status			
Married	0.29	0.29	*0.29*
Living with partner	0.21	0.21	*0.21*
Not living with partner	0.50	0.50	*0.51*
Has children	0.48	0.47	*0.43*
**Work characteristics**			
Job tenure			
Two years or less	0.49	0.49	*0.49*
Three to five years	0.22	0.23	*0.23*
Six or more years	0.28	0.28	*0.28*
Manager	0.25	0.26	*0.33*
Union member	0.08	0.08	*0.07*
Schedule stability (last month)			
At least two weeks' notice	0.40	0.39	*0.36*
Canceled shift	0.13	0.13	*0.15*
On-call shift	0.24	0.24	*0.27*
Shift timing changed	0.63	0.63	*0.65*
Usual weekly work hours	33.7	33.9	*34.7*
Treated	0.05	0.05	*0.03*
N	87123	99482	*12359*

Table S7. Comparison of Characteristics of Analysis Sample Between Geographic Assignment Approaches. Alternative geographic assignment: using IP address to fill in gaps in self-reported location information.

	Control			Treatment			Diff.-in-Diff.
	Pre	Post	Diff.	Pre	Post	Diff.	
Two weeks' notice	0.35	0.35	0.00	0.45	0.60	0.15	0.15
Clopening	0.44	0.40	-0.04	0.45	0.31	-0.14	-0.10
Timing change	0.66	0.65	-0.01	0.65	0.57	-0.08	-0.07
Canceled shift	0.13	0.13	0.00	0.15	0.13	-0.02	-0.02
On-call shift	0.27	0.26	-0.01	0.24	0.23	-0.01	0.00
Usual hours	33.02	34.05	1.03	33.27	33.31	0.04	-0.99
N	124888	156963	281851	1585	2557	4142	285993

Table S8. Comparison of Scheduling Outcomes Between Treatment & Control Group in Pre- & Post-Periods. Weighted using equal sub-experiment weights.

	Control			Treatment			Diff.-in-Diff.
	Pre	Post	Diff.	Pre	Post	Diff.	
**Seattle**							
Two weeks' notice	0.41	0.38	-0.03	0.60	0.63	0.03	0.06
Clopening	0.44	0.45	0.01	0.33	0.31	-0.02	-0.03
Timing change	0.66	0.65	-0.01	0.64	0.61	-0.03	-0.02
Canceled shift	0.14	0.14	0.00	0.14	0.17	0.03	0.03
On-call shift	0.27	0.24	-0.03	0.19	0.20	0.01	0.04
Usual hours	31.85	33.44	1.59	32.31	32.08	-0.23	-1.82
N	8227	43548	51775	275	604	879	52654
**Oregon**							
Two weeks' notice	0.37	0.39	0.02	0.46	0.62	0.16	0.14
Clopening	0.46	0.41	-0.05	0.40	0.31	-0.09	-0.04
Timing change	0.65	0.63	-0.02	0.66	0.65	-0.01	0.01
Canceled shift	0.13	0.14	0.01	0.11	0.11	0.00	-0.01
On-call shift	0.25	0.24	-0.01	0.21	0.25	0.04	0.05
Usual hours	33.03	33.65	0.62	33.05	32.93	-0.12	-0.74
N	25502	36963	62465	827	1684	2511	64976
**New York City**							
Two weeks' notice	0.21	0.21	0.00	0.38	0.66	0.28	0.28
Clopening	0.41	0.41	0.00	0.48	0.27	-0.21	-0.21
Timing change	0.70	0.75	0.05	0.70	0.60	-0.10	-0.15
Canceled shift	0.13	0.14	0.01	0.20	0.13	-0.07	-0.08
On-call shift	0.35	0.35	0.00	0.31	0.32	0.01	0.01
Usual hours	33.68	33.87	0.19	32.04	30.54	-1.50	-1.69
N	6917	9903	16820	104	108	212	17032
**Philadelphia**							
Two weeks' notice	0.39	0.38	-0.01	0.36	0.44	0.08	0.09
Clopening	0.46	0.37	-0.09	0.53	0.29	-0.24	-0.15
Timing change	0.65	0.60	-0.05	0.64	0.47	-0.17	-0.12
Canceled shift	0.13	0.13	0.00	0.15	0.05	-0.10	-0.10
On-call shift	0.24	0.24	0.00	0.26	0.15	-0.11	-0.11
Usual hours	32.98	34.61	1.63	35.52	38.09	2.57	0.94
N	40166	35904	76070	170	73	243	76313
**Chicago**							
Two weeks' notice	0.38	0.39	0.01	0.45	0.66	0.21	0.20
Clopening	0.44	0.37	-0.07	0.50	0.38	-0.12	-0.05
Timing change	0.64	0.61	-0.03	0.61	0.55	-0.06	-0.03
Canceled shift	0.14	0.11	-0.03	0.16	0.16	0.00	0.03
On-call shift	0.24	0.23	-0.01	0.25	0.23	-0.02	-0.01
Usual hours	33.50	34.68	1.18	33.41	32.93	-0.48	-1.66
N	44076	30645	74721	209	88	297	75018

Table S9. Comparison of Scheduling Outcomes Between Treatment & Control Group in Pre- & Post-Periods, by Jurisdiction.

	(1)	(2)	(3)
Two weeks' notice			
FWW × Post / ATT	0.130***	0.091***	0.120***
	(0.032)	(0.019)	(0.032)
Clopening			
FWW × Post / ATT	-0.084***	-0.049***	-0.050
	(0.025)	(0.010)	(0.034)
Timing change			
FWW × Post / ATT	-0.067*	-0.049*	0.008
	(0.029)	(0.021)	(0.033)
Canceled shift			
FWW × Post / ATT	-0.026	-0.015	0.017
	(0.021)	(0.017)	(0.023)
On-call shift			
FWW × Post / ATT	-0.013	-0.010	0.052+
	(0.019)	(0.019)	(0.029)
Usual hours			
FWW × Post / ATT	-0.373	-0.226	-1.141
	(0.723)	(0.720)	(0.751)
N	285993	327944	87391
Employer (× sub-experiment) FE	X	X	X
Individual-level controls	X	X	X
Area-level controls	X	X	X
Wing et al. (2024) model	X	X	
Callaway-Sant'Anna (2021) model			X
All industries inc. in comp. groups		X	X

Table S10. Effects of Fair Work Week Laws, Robustness to Callaway-Sant'Anna Model. + $p < 0.10$, * $p < 0.05$, ** $p < 0.01$, *** $p < 0.001$. M1 and M2 are weighted using equal sub-experiment weights and have standard errors clustered at the geographic level.
